# Trends in dermatomyositis- and polymyositis-related mortality in the state of São Paulo, Brazil, 1985-2007: multiple cause-of-death analysis

**DOI:** 10.1186/1471-2458-10-597

**Published:** 2010-10-11

**Authors:** Augusto H Santo, José Maria P Souza, Celso E Pinheiro, Deborah CC Souza, Emilia I Sato

**Affiliations:** 1 Faculdade de Saúde Pública da Universidade de São Paulo, Brazil; 2 Departamento de Informática do Sistema Único de Saúde, Ministério da Saúde, Brazil; 3 Escola Paulista de Medicina, Universidade Federal de São Paulo, São Paulo, Brazil

## Abstract

**Background:**

Dermatomyositis (DM) and polymyositis (PM) are rare systemic autoimmune rheumatic diseases with high fatality rates. There have been few population-based mortality studies of dermatomyositis and polymyositis in the world, and none have been conducted in Brazil. The objective of the present study was to employ multiple-cause-of-death methodology in the analysis of trends in mortality related to dermatomyositis and polymyositis in the state of São Paulo, Brazil, between 1985 and 2007.

**Methods:**

We analyzed mortality data from the São Paulo State Data Analysis System, selecting all death certificates on which DM or PM was listed as a cause of death. The variables sex, age and underlying, associated or total mentions of causes of death were studied using mortality rates, proportions and historical trends. Statistical analysis were performed by chi-square and H Kruskal-Wallis tests, variance analysis and linear regression. A p value less than 0.05 was regarded as significant.

**Results:**

Over a 23-year period, there were 318 DM-related deaths and 316 PM-related deaths. Overall, DM/PM was designated as an underlying cause in 55.2% and as an associated cause in 44.8%; among 634 total deaths females accounted for 71.5%. During the study period, age- and gender-adjusted DM mortality rates did not change significantly, although PM as an underlying cause and total mentions of PM trended lower (p < 0.05). The mean ages at death were 47.76 ± 20.81 years for DM and 54.24 ± 17.94 years for PM (p = 0.0003). For DM/PM, respectively, as underlying causes, the principal associated causes of death were as follows: pneumonia (in 43.8%/33.5%); respiratory failure (in 34.4%/32.3%); interstitial pulmonary diseases and other pulmonary conditions (in 28.9%/17.6%); and septicemia (in 22.8%/15.9%). For DM/PM, respectively, as associated causes, the following were the principal underlying causes of death: respiratory disorders (in 28.3%/26.0%); circulatory disorders (in 17.4%/20.5%); neoplasms (in 16.7%/13.7%); infectious and parasitic diseases (in 11.6%/9.6%); and gastrointestinal disorders (in 8.0%/4.8%). Of the 318 DM-related deaths, 36 involved neoplasms, compared with 20 of the 316 PM-related deaths (p = 0.03).

**Conclusions:**

Our study using multiple cause of deaths found that DM/PM were identified as the underlying cause of death in only 55.2% of the deaths, indicating that both diseases were underestimated in the primary mortality statistics. We observed a predominance of deaths in women and in older individuals, as well as a trend toward stability in the mortality rates. We have confirmed that the risk of death is greater when either disease is accompanied by neoplasm, albeit to lesser degree in individuals with PM. The investigation of the underlying and associated causes of death related to DM/PM broaden the knowledge of the natural history of both diseases and could help integrate mortality data for use in the evaluation of control measures for DM/PM.

## Background

Dermatomyositis and polymyositis are rare systemic autoimmune rheumatic diseases characterized by chronic inflammatory myopathies. Both diseases involve an immune process that is triggered by environmental factors in genetically susceptible individuals, although the etiopathogenesis remains unknown [1]. Before modern treatments became available, the fatality related to these conditions was as high as 50-70% [2]. Currently reported fatality ratios vary widely--from 2% to 45% [3,4].

Studies on the impact of rheumatic diseases should include mortality data as a complement to those regarding functional status, work capacity and overall quality of life [5]. One limitation of the majority of studies on mortality in myositis is that they involve cohorts that are small or are followed at single medical facilities, and there have been few population-based studies on the topic [4]. Only specialized facilities accumulate a sufficient number of patients to analyze the mortality related to diseases such as polymyositis [6]. The analysis of mortality statistics was considered the principal means of determining the frequency of dermatomyositis and other connective tissue diseases [7]. A study conducted in Finland demonstrated that mortality rates were three times higher in patients with dermatomyositis or polymyositis than in a group of age- and gender-matched individuals of the population [4,8]. In the Netherlands, the 5-year risk of death for myositis patients was shown to be 11%, compared with 1-2% for individuals of the same mean age of the Dutch population [3,4].

In Brazil, mortality statistics are derived from death certificates, which are provided by physicians or are created based on witness statements, requiring notarization in either case [9-11]. Copies of the death certificates are sent to the state bureaus of vital statistics or departments of epidemiological surveillance, where the demographic and medical data are encoded and processed, the resulting data sets then being forwarded to the Brazilian Ministry of Health for consolidation at the national level [12]. Among those data, considerable importance is given to the causes of death. The underlying cause of death is defined as "*(a) the disease or injury which initiated the train of morbid events leading directly to death; or (b) the circumstances of the accident or violence which produced the fatal injury*" [13]. With the objective of preventing deaths, the underlying cause is presented in the so-called primary mortality statistics. Despite the value of knowing the underlying cause, there has been, since the mid-20th century, an increasing demand for taking into account all of the causes of death listed on death certificates. This methodology, designated multiple cause of death analysis, furnishes information regarding all of the conditions and circumstances involved in the process that culminated in death, offering new elements and perspectives for prevention [14,15]. Multiple cause of death analysis is particularly recommended for the study of the mortality in chronic diseases, such as rheumatic diseases [16,17]. Data related to multiple causes of death have been available for the state of São Paulo since 1983 and for Brazil as a whole since 1999 [14,18,19].

The objective of the present study was to employ multiple cause of death analysis in the study of trends in mortality related to dermatomyositis and polymyositis in the state of São Paulo between 1985 and 2007.

## Methods

The annual mortality data were obtained from the publicly available databases of the *Sistema Estadual de Análise de Dados *(SEADE, State Data Analysis System) Foundation, the organ responsible for vital statistics, operating under the auspices of the São Paulo State Secretary of Economics and Planning. We selected all deaths in which dermatomyositis or polymyositis were listed on any line or in either part of the International Form of Medical Certificate of Cause of Death (the medical certification section of the death certificate), whether categorized as the underlying cause of death or as an associated (non-underlying) cause. Complications of the underlying cause (Part I of the medical certification section) and contributing causes (Part II of the medical certification section) were collectively designated associated causes of death. We employed the 1985-2007 annual population estimates for the state of São Paulo, which were recently updated by the SEADE Foundation [20]. Those data, based on the 2007 Census, reveal trends in the demographic components of the vital statistics (updated through 2007) and the changes in population growth.

We selected all deaths in which one of the following subcategories of the ninth or tenth revision of the International Classification of Diseases (ICD-9 or ICD-10) were listed [21,22]: 710.3 (ICD-9, Dermatomyositis); 710.4 (ICD-9, Polymyositis); M33.0 (ICD-10, Juvenile dermatomyositis); M33.1 (ICD-10, Other dermatomyositis); M33.2 (ICD-10, Polymyositis); and M33.9 (ICD-10, Dermatopolymyositis, unspecified). Equivalency tables were constructed in order to compare the ICD-9 and ICD-10 categories in terms of the trends in the causes of death [23,24]. In order to reconstruct the morbid process leading to death, all of the causes of death listed in the medical certification section of the death certificate were considered, even those classified as ill-defined and those characterized by the World Health Organization (WHO) as modes of death [13].

The records included in the mortality databases contain fields that mirror those appearing on the official Brazilian death certificate. In the present study, it was necessary to standardize the structure of the records studied, since various fields on the death certificate had been modified, in terms of designation, size and the codes used in order to identify variables, between 1985 and 2007. In addition, we created auxiliary fields for the study of multiple causes, including a field designed to contain a single "string" of characters composed of the codes entered on lines (a), (b), (c) and (d) of Parts I and of Part II of the medical certification section of the death certificate.

Between 1985 and 1995, the automatic processing of data related to the causes of death was performed with the Automated Classification of Medical Entities (ACME) program [14,15,19,25-27], after which (between 1996 and 2007) it was performed with the *Declarações de Óbito de São Paulo *(DOSP, São Paulo Death Certificates) program [28], an adaptation that allowed the data to be processed in batches using the Underlying Cause Selection program [19]. The data regarding the associated causes of death in 1996 are incomplete due to the fact that the SEADE Foundation implemented the use of the DOSP in April of that year. The automatic processing involves the use of algorithms and decision tables that incorporate the WHO mortality standards and the etiological relationships among the causes of death. The ACME decision tables [29,30] are considered *de facto *international standards for the automatic processing of data related to the causes of death [17,31]. The structural difference between the numeric codes employed in the ICD-9 (used up through 1995) and the alphanumeric codes employed in the ICD-10 (in use since 1996) affected the processing of the data in that a different program was required for each coding system. As a consequence, we studied the trends in mortality related to dermatomyositis or polymyositis in two separate periods: 1985-1995 and 1996-2007. The expressions "death from" and "death due to" refer to the underlying cause of death, whereas "deaths with a mention of" and "mortality related to" refer to the listing of a given condition either as the underlying cause or as an associated cause. The causes of death evaluated in the present study were those originally mentioned in the medical certification section, internationally classified as "*entity axis codes"*, which were defined and presented under the structure and headings of the ICD [14,32].

Using mortality rates, proportions and historical trends, we studied the distributions of the following variables: gender; age at death; year of death; underlying cause of death; associated cause(s) of death; total mentions of each cause of death; and mean number of causes listed per death certificate. Medical and demographic variables were processed with the following programs: dBASE III Plus, version 1.1 (Ashton-Tate Corporation, Torrance, CA), Epi Info, version 6.04d (Centers for Disease Control and Prevention, Atlanta, GA), Excel 2007 (Microsoft Corporation, Redmond, WA) and Stata/IC for Windows, version 10 (Stata Corp LP, College Station, TX). We used the *Tabulador de Causas Múltiplas *(Multiple Causes Tabulator) program (DATASUS, Ministério da Saúde, Faculdade de Saúde Pública, Universidade de São Paulo, Brazil), available for ICD-9 and ICD-10 (TCM-9, version 4.0; and TCM-10, version 2.2), in our presentation of the associated causes and of the mean number of causes per death certificate [33]. For the presentation of the associated causes listed on the death certificates on which dermatomyositis or polymyositis were identified as the underlying cause, we prepared special lists showing the causes involved in the respective natural histories [1,34-38], as well as those mentioned with the greatest frequency. The duplication or multiplication of causes of death was avoided when the causes were presented in abbreviated lists. The number of causes depends on the breadth of the class (subcategory, category, grouping or chapter of the ICD-9 or ICD-10). Therefore, if two or more causes mentioned in the medical certification section were included in the same class, only one cause was computed [14,33]. The *Separador de Registros de Mortalidade *(SRM_DBF, Mortality Records Separator_dBASE File) program (DATASUS, Ministério da Saúde, Faculdade de Saúde Pública, Universidade de São Paulo, Brazil), with versions for ICD-9 and ICD-10 (version 1.1 and version 3.1, respectively), was used in order to compile records in which there was interest in specific associated causes of death. The study of the joint mention among the causes of death listed on the death certificates was performed with the *Tabulador de Causas Conjuntas *(Joint Causes Tabulator, version 1.3, for ICD-9 and ICD-10) program. The ratio between the observed number of cause-of-death pairs and the expected number of such pairs, under the hypothesis of independence was calculated [14,15,17].

Mortality rates (per 1,000,000 population) for dermatomyositis and polymyositis were calculated--by year, by subperiod and for the study period (1985-2007) as a whole--based on the number of deaths in which each disease had been identified as the underlying cause or as an associated cause, as well as on the total number of mentions of each disease. In order to calculate the mean mortality rate for each subperiod studied, the number of deaths occurring in the period was divided by the sum of the respective annual population counts, analogous to the procedure used in order to calculate the mean mortality rate for the 23-year study period as a whole. The *Análisis Epidemiológico de Datos Tabulados *(Epidat, Epidemiological Analysis of Tabulated Data) program, version 3.1 (Pan American Health Organization; http://dxsp.sergas.es) was used for standardize, by the direct method, the age-adjusted crude and mean mortality rates, by subperiod and for the study period as a whole, to the population of Brazil in 2000.

### Statistical analysis

We used the chi-square test in order to analyze joint mentions and the associations between causes of death; analysis of variance in order to compare the mean numbers of causes mentioned on the death certificate; the Kruskal-Wallis H test in order to compare the mean age at death between groups; and linear regression in order to evaluate the magnitude and significance of historical trends in the mean ages at death and the standardized mortality rates. Values of p < 0.05 were considered significant.

## Results

In the state of São Paulo, there were 318 and 316 deaths related to dermatomyositis and polymyositis, respectively, between 1985 and 2007, totaling 634 deaths (annual mean, 27.6 deaths). Among those 634 deaths, one of the two diseases was listed as the underlying cause in 350 (55.2%) and as an associated cause of death in 284 (44.8%), resulting in annual means of 15.2 and 12.3 deaths, respectively. There was a marked annual variation in the total number of dermatomyositis- or polymyositis-related deaths, which ranged from 20 (in 1986) to 43 (in 1999), although the trend was to remain close to the mean number. Death related to dermatomyositis or polymyositis was more common among women, who accounted for 453 (71.5%) of the 634 deaths. The majority (65.8%) of the dermatomyositis- or polymyositis-related deaths occurred in individuals over 45 years of age, particularly in those between 55 and 64 years of age, who accounted for 22.2% of such deaths (Table 1)

**Table 1:** Mortality related to dermatomyositis and polymyositis, according to the qualification of the cause-of-death, absolute numbers and percentage of deaths, gender and age mean, crude, standardized and specific death rates, mean, median and mode of the ages at death, state of São Paulo, Brazil. 1985 to 2007.

	Dermatomyositis			Polymyositis			Total mentions		
	Underlying	Associated	Mentions	Underlying	Associated	Mentions	Underlying	Associated	Mentions
GENDER. ABSOLUTE NUMBERS (%)									
Male	41 (22.8)	42 (30.4)	83 (26.1)	57 (33.5)	41 (28.1)	98 (31.0)	98 (28.0)	83 (29.2)	181 (28.5)
Female	139 (77.2)	96 (69.6)	235 (73.9)	113 (66.5)	105 (71.9)	218 (69.0)	252 (72.0)	201 (70.8)	453 (71.5)
Total	180 (100.0)	138 (100.0)	318 (100.0)	170 (100.0)	146 (100.0)	316 (100.0)	350 (100.0)	284 (100.0)	634 (100.0)
									
MEAN CRUDE SPECIFIC DEATH RATES (PER 1.000.000 POPULATION) ACCORDING TO GENDER									
Male	0.1054	0.1079	0.2133	0.1465	0.1054	0.2519	0.2519	0.2133	0.4652
Female	0.3460	0.2389	0.5849	0.2813	0.2614	0.5426	0.6272	0.5003	1.1275
Total	0.2276	0.1745	0.4021	0.2150	0.1846	0.3996	0.4426	0.3591	0.8017
									
MEAN STANDARDIZED SPECIFIC DEATH RATES (PER 1.000.000 POPULATION) ACCORDING TO GENDER									
Male	0.1074	0.1082	0.2155	0.1494	0.1079	0.2572	0.2567	0.2160	0.4728
Female	0.3299	0.2249	0.5548	0.2629	0.2421	0.5050	0.5928	0.4670	1.0598
Total	0.2232	0.1705	0.3936	0.2096	0.1807	0.3902	0.4327	0.3511	0.7839
Ratio F/M	3.07	2.08	2.57	1.76	2.24	1.96	2.31	2.16	2.24
									
AGE IN YEARS. ABSOLUTE NUMBERS (%)									
00-14	24 (13.3)	10 (7.2)	34 (10.7)	5 (2.9)	4 (2.7)	9 (2.8)	29 (8.3)	14 (4.9)	43 (6.8)
15-24	14 (7.8)	9 (6.5)	23 (7.2)	9 (5.3)	3 (2.1)	12 (3.8)	23 (6.6)	12 (4.2)	35 (5.5)
25-34	15 (8.3)	11 (8.00)	26 (8.2)	16 (9.4)	11 (7.5)	27 (8.5)	31 (8.9)	22 (7.7)	53 (8.4)
35-44	22 (12.2)	17 (12.3)	39 (12.3)	25 (14.7)	22 (15.1)	47 (14.9)	47 (13.4)	39 (13.7)	86 (13.6)
45-54	36 (20.0)	20 (14.5)	56 (17.6)	29 (17.1)	26 (17.8)	55 (17.4)	65 (18.6)	46 (16.2)	111 (17.5)
55-64	36 (20.0)	37 (26.8)	73 (23.0)	41 (24.1)	27 (18.5)	68 (21.5)	77 (22.0)	64 (22.5)	141 (22.2)
65-74	22 (12.2)	22 (15.9)	44 (13.8)	24 (14.1)	32 (21.9)	56 (17.7)	46 (13.1)	54 (19.0)	100 (15.8)
≥75	11 (6.1)	12 (8.7)	23 (7.2)	21 (12.4)	21 (14.4)	42 (13.3)	32 (9.1)	33 (11.6)	65 (10.3)
Total	180 (100.0)	138 (100.0)	318 (100.0)	170 (100.0)	146 (100.0)	316 (100.0)	350 (100.0)	284 (100.0)	634 (100.0)
									
MEAN SPECIFIC DEATH RATES (PER 1.000.000 POPULATION) ACCORDING TO AGE									
00-14	0.1088	0.0453	0.1541	0.0227	0.0181	0.0408	0.1315	0.0635	0.1950
15-24	0.0939	0.0603	0.1542	0.0603	0.0201	0.0805	0.1542	0.0805	0.2347
25-34	0.1097	0.0804	0.1901	0.1170	0.0804	0.1974	0.2267	0.1609	0.3875
35-44	0.1979	0.1529	0.3509	0.2249	0.1979	0.4228	0.4228	0.3509	0.7737
45-54	0.4615	0.2564	0.7180	0.3718	0.3333	0.7051	0.8333	0.5897	1.4231
55-64	0.7185	0.7384	1.4569	0.8182	0.5388	1.3571	1.5367	1.2773	2.8140
65-74	0.7420	0.7420	1.4841	0.8095	1.0793	1.8888	1.5515	1.8214	3.3729
≥75	0.7113	0.7760	1.4873	1.3580	1.3580	2.7160	2.0693	2.1340	4.2033
									
AGES AT DEATH (YEARS)									
Mean (SD)	45.40 (± 21.20)	50.84 (± 19.95)	47.76 (± 20.81)	52.68 (± 18.41)	56.06 (± 17.25)	54.24 (± 17.94)	48.93 (± 20.20)	53.52 (± 18.76)	50.99 (± 19.69)
Median	49.00	56.00	51.50	55.50	57.50	56.50	52.50	57.00	54.50
Mode	52.50	64.50	64.50	61.50	61.50	61.50	42.50	61.50	61.50

Source: Fundação Sistema Estadual de Análise de Dados (SEADE)

**Figure 1:**
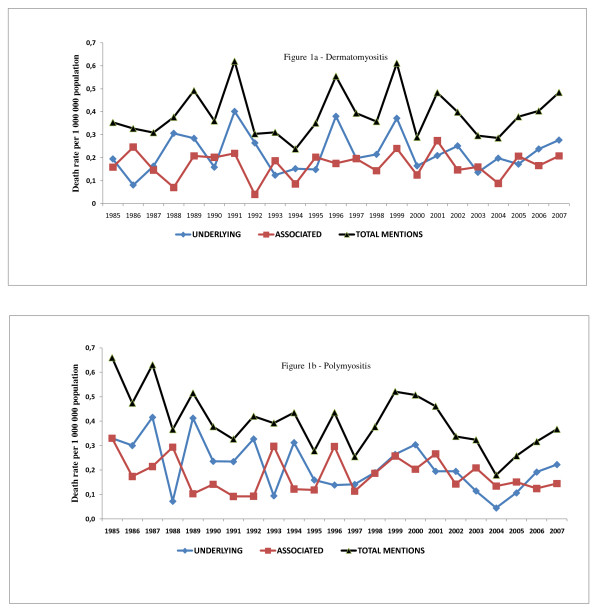
**Trends of death rates of dermatomyositis and polymyositis, underlying, associated and total causes of death, State of São Paulo, Brazil, 1985 to 2007**.

The linear regression showed that, in the period studied, there were no significant variations in the standardized mortality rates for dermatomyositis as an underlying cause (p = 0.786), as an associated cause (p = 0.741) or in terms of the total mentions (p = 0.684); nor was there any such variation in the standardized mortality rates for polymyositis as an associated cause of death (p = 0.340). However, for the deaths related to polymyositis, we observed significant decreases in the standardized mortality rates, for polymyositis as the underlying cause (p = 0.016), which fell from 0.3297/1,000,000 population in 1985 to 0.2225/1,000,000 population in 2007, as well as for the total mentions of polymyositis (p = 0.005), which fell from 0.6597/1,000,000 population in 1985 to 0.3669/1,000,000 population in 2007. Figures 1a and 1b show the trends in the standardized mortality rates according to the causes of death in dermatomyositis- or polymyositis-related deaths.

The female/male ratio for the standardized mean mortality rates in the 23-year study period as a whole was 2.24:1 for all of the deaths studied, ranging from 3.07:1 for dermatomyositis as the underlying cause to 1.76:1 for polymyositis, as the underlying cause (Table 1). Although the overall distribution between the genders in terms of the underlying cause and associated causes was similar to that observed for all of the deaths studied, we found dermatomyositis as the underlying cause of death to be more common among women, who accounted for 139 (59.1%) of the 235 such deaths, whereas men were more often the victims of fatal polymyositis, accounting for 57 (58.2%) of the 98 deaths in which polymyositis was listed as the underlying cause (Table 1).

**Figure 2:**
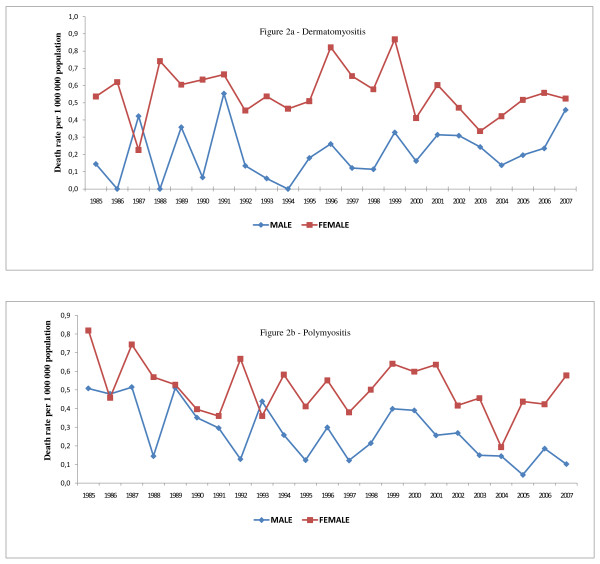
**Trends of death rates of dermatomyositis and polymyositis according to gender, State of São Paulo, Brazil, 1985 to 2007**.

The linear regression revealed no significant variations in the standardized mortality rates by total mentions, of dermatomyositis--in men (p = 0.267) or in women (p = 0.575)--or of polymyositis in women (p = 0.102), over the course of the study period. However, there was a significant decrease in the standardized mortality rate, by total mentions, for polymyositis in men (p = 0.001), which fell from 0.5078/1,000,000 population in 1985 to 0.1025/1,000,000 population in 2007 (Figures 2a and 2b).

Considering the 23-year study period as a whole, we found that the specific dermatomyositis- and polymyositis-related mortality rates were directly proportional to the age group studied, following a unimodal distribution. Consequently, the highest mortality rates were found in the ≥ 75-year age bracket, except for dermatomyositis or polymyositis as the underlying cause in men, for which the highest mortality rates were in the 65- to 74-year age bracket. In the ≤ 24-year age bracket, the underlying cause of death was dermatomyositis in 67% (38 of the 57 deaths studied), as was also true for polymyositis (14 of the 21 deaths studied), compared with 54% (142 of the 261 deaths studied) and 53% (156 of the 295 deaths studied), respectively, in the ≥ 25-year age bracket (Table 1).

Despite being influenced by the qualification of the cause of death, the mean ages at death were consistently lower among the deaths related to dermatomyositis than among those related to polymyositis: as the underlying cause--45.40 ± 21.20 and 52.68 ± 18.41 years, respectively (p = 0.002822); as an associated cause--50.84 ± 19.95 and 56.06 ± 17.25 years, respectively (p = 0.039359); and as a mentioned cause--47.76 ± 20.81 and 54.24 ± 17.94 years, respectively (p = 0.000279). Likewise, the median and mode ages were lower among the deaths related to dermatomyositis than among those related to polymyositis (Table 1).

The mean ages at death were also higher among women than among men, whether death was attributed to dermatomyositis (48.06 ± 20.98 vs. 46.89 ± 20.42 years) or to polymyositis (54.61 ± 18.20 vs. 53.42 ± 17.42), although the differences were not significant.

The linear regression showed that there were no significant differences between 1985 and 2007 in terms of the mean ages at death among the dermatomyositis-related deaths in men and women (p = 0.172 and p = 0.081, respectively) or among the polymyositis-related deaths in men (p = 0.538). Among the polymyositis-related deaths in women, there was a temporal increase in the age at death (p = 0.004), which rose from 45.32 ± 20.52 years in 1985 to 58.10 ± 20.06) years in 2007.

For the 23-year study period as a whole, the following were the principal associated causes of death when the underlying cause was identified as dermatomyositis or polymyositis, respectively: pneumonia--in 43.3% and 33.5%; respiratory failure--in 34.4% and 32.4%; interstitial lung disease and other respiratory diseases--in 28.9% and 17.6% (p = 0.013074); and septicemia--in 22.8% and 15.9%. Neoplasm occurred as an associated cause only in the deaths attributed to dermatomyositis. (Table 2)

**Table 2:** Associated (non-underlying) causes in deaths related to dermatomyositis and polymyositis as an underlying cause, state of São Paulo, 1985 to 2007

Associated (non-underlying) causes of death (ICD-9) (ICD-10)	Dermatomyositis		Polymyositis	
	(deaths = 180)		(deaths = 170)	
	n	%	n	%
Pneumonia (480.0-483, 485-486, 507.0) (J12.0-J16.8, J18.0-J18.9, J69.0)	78	43.3	57	33.5
Respiratory failure (7860) (J96)	62	34.4	55	32.4
Interstitial pulmonary disease and other pulmonary diseases (510-519) (J80-J95, J98)	52	28.9	30	17.6
Septicaemia (038.0-038.9) (A40.0-A41.9)	41	22.8	27	15.9
Hypertensive diseases and other heart diseases (401-427, 429) (I10-I49, I51)	21	11.7	21	12.4
Multiple organs failure (7998) (R688)	22	12.2	17	10.0
Heart failure (428.0-428.9) (I50.0-I50.9)	13	7.2	21	12.4
Cardiorespiratory failure (427.5, 799.1) (I46.9, R092)	11	6.1	22	12.9
Diabetes mellitus (250.0-250.9) (E10.0-E14.9)	15	8.3	11	6.5
Shock (785.5) (R57.0-R57.9)	13	7.2	11	6.5
Renal failure (584.5-586) (N17.0-N19)	5	2.8	15	8.8
Malnutrition/Cachexia (260.0-263.9, 799.4) (E40-E46, R64)	6	3.3	11	6.5
Neoplasms (140.0-239.9) (C00.0-D48.9)	13	7.2	0	0.0
Remaining associated causes of death	52	28.9	49	28.8

Source: Fundação Sistema Estadual de Análise de Dados (SEADE)

Percents calculated in relation to the number of deaths

Rubrics and codes of the International Classification of Diseases, Injuries and Causes of Death, Ninth Revision (1985 to 1995)

Rubrics and codes of the International Statistical Classification of Diseases and Related Health Problems, Tenth Revision (1996 to 2007)

Data for 1996 are partial

As shown in Table 3, the principal underlying causes of death when dermatomyositis or polymyositis was listed as an associated cause were, respectively, as follows: diseases of the respiratory system--in 29.0% and 26.0%; diseases of the circulatory system--in 17.4% and 20.5%; neoplasms--in 15.9% and 13.7%; certain infectious and parasitic diseases--in 11.6% and 9.6%; diseases of the digestive system--in 8.0% and 4.8%; and endocrine, nutritional and metabolic disorders--in 4.3% and 8.9%.

**Table 3:** Underlying causes in deaths related to dermatomyositis and polymyositis as an associated cause, state of São Paulo, 1985 to 2007

Underlying causes of death (ICD-9) (ICD-10)	Dermatomyositis		Polymyositis		Total	
	
	n	%	n	%	n	%
**Certain infectious and parasitic diseases (001-139) (A00-B99)**	**16**	**11.6**	**14**	**9.6**	**30**	**10.6**
Tuberculosis (010-018) (A15-A19)	5	3.6	1	0.7	6	2.1
Septicaemia (038) (A40-A41)	4	2.9	4	2.7	8	2.8
Human immunodeficiency virus {HIV} disease (279.1) (B20-B24)	1	0.7	4	2.7	5	1.8
**Neoplasms (140-239) (C00-D48)**	**23**	**16.7**	**20**	**13.7**	**43**	**15.1**
**Diseases of the blood and blood-forming organs and ... (280-289) (D50-D89)**	**8**	**5.8**	**5**	**3.4**	**13**	**4.6**
Certain disorders involving the immune mechanism (279.0, 279.2-279,9) (D80-D89)	7	5.1	2	1.4	9	3.2
**Endocrine, nutritional and metabolic diseases (240-278) (E00-E90)**	**6**	**4.3**	**13**	**8.9**	**19**	**6.7**
Diabetes mellitus (250) (E10-E14)	1	0.7	8	5.5	9	3.2
**Diseases of the nervous system (320-359) (G00-G98)**	**2**	**1.4**	**2**	**1.4**	**4**	**1.4**
**Diseases of the circulatory system (390-459) (I00-I99)**	**24**	**17.4**	**30**	**20.5**	**54**	**19.0**
Hypertensive diseases (401-404) (I10-I13)	0	0.0	3	2.1	3	1.1
Ischaemic heart diseases (410-414) (I20-I25)	7	5.1	10	6.8	17	6.0
Cardiomyopathy (425) (I42)	4	2.9	4	2.7	8	2.8
Cerebrovascular diseases (430-438) (I60-I69)	1	0.7	5	3.4	6	2.1
Arteritis, phlebitis and thrombophlebitis (447.6, 451) (I77.6, I80)	0	0.0	1	0.7	1	0.4
**Diseases of the respiratory system (460-519) (J00-J99)**	**39**	**28.3**	**38**	**26.0**	**77**	**27.1**
Pneumonia (480-486) (J12-J18)	21	15.2	24	16.4	45	15.8
**Diseases of the digestive system (520-579) (K00-K93)**	**11**	**8.0**	**7**	**4.8**	**18**	**6.3**
**Diseases of the skin and subcutaneous tissue (680-709) (L00-L99)**	**1**	**0.7**	**1**	**0.7**	**2**	**0.7**
**Diseases of the musculoskeletal system and connective tissue (710-739) (M00-M99)**	**5**	**3.6**	**10**	**6.8**	**15**	**5.3**
Pyogenic arthritis (711.0) (M00)	0	0.0	1	0.7	1	0.4
Systemic lupus erythematosus (710.0) (M32)	5	3.6	3	2.1	8	2.8
Systemic sclerosis (710.1) (M34)	0	0.0	5	3.4	5	1.8
Pathological fracture (733.1) (M84.4)	0	0.0	1	0.7	1	0.4
**Diseases of the genitourinary system (580-629) (N00-N99)**	**3**	**2.2**	**6**	**4.1**	**9**	**3.2**
Renal failure (584-586) (N17-N19)	0	0.0	3	2.1	3	1.1

**TOTAL**	**138**	**100.0**	**146**	**100.0**	**284**	**100.0**

Source: Fundação Sistema Estadual de Análise de Dados (SEADE)

Rubrics and codes of the International Classification of Diseases, Injuries and Causes of Death, Ninth Revision (1985 to 1995)

Rubrics and codes of the International Statistical Classification of Diseases and Related Health Problems, Tenth Revision (1996 to 2007)

In addition to the associations described above for dermatomyositis and polymyositis as underlying or associated causes, it is of note that, of the 634 death certificates, there were joint mentions of pneumonia and septicemia on 84 (observed/expected ratio, 1.62) and of pneumonia and respiratory failure on 73 (observed/expected ratio, 1.41). Similarly, dermatomyositis and polymyositis were mentioned jointly with systemic lupus erythematosus on 5 and 8 of the death certificates, respectively, systemic sclerosis on 3 and 9, respectively, diffuse connective tissue diseases on 2 and 1, respectively, and muscle disorders on 1 and 2, respectively. In addition, dermatomyositis was mentioned jointly with polymyositis and with juvenile chronic polyarthritis on 1 death certificate each, whereas polymyositis was mentioned jointly with rheumatoid arthritis on 8, with non-specific arthritis on 1 and with scoliosis on 1.

For the two subperiods studied (1985-1995 and 1996-2007), the mean number of causes of death per death certificate among those listing dermatomyositis or polymyositis as the underlying cause was 2.98 ± 0.81 and 3.49 ± 1.21, respectively, compared with 3.90 ± 1.01 and 4.22 ± 1.05 among those listing either disease as an associated cause. All of the differences between these means, two by two, between the periods and between the qualifications of the causes of death, were significant (p < 0.05).

Considering all of the causes of death listed, we found that neoplasm co-occurred in 36 of the 318 deaths related to dermatomyositis and in 20 of the 316 deaths related to polymyositis. Therefore, the probability of neoplasm was greater when death was related to dermatomyositis (**χ^2 ^**= 4.90; p = 0.026790), corresponding to a relative risk of 1.79 (range, 1.07-3.04) between the respective specific standardized mortality rates. In 1996, the ICD-10 was introduced and juvenile dermatomyositis came to be classified separately in the subcategory M33.0. Between 1996 and 2007, there were no joint mentions of juvenile dermatomyositis and neoplasm on any of the death certificates included in the present study. The proportion of women was greater among the deaths in which there was a joint mention of neoplasm and dermatomyositis than among those in which there was a joint mention of neoplasm and polymyositis, the female/male ratios being 3.5 and 1.5, respectively, although the differences were not significant. Of the 56 deaths in which neoplasm was mentioned jointly with dermatomyositis or polymyositis, 49 (87.5%) occurred in individuals ≥ 45 years of age. The mean age at death among the deaths in which neoplasm was mentioned jointly with dermatomyositis was of 61.50 ± 13.66 years, significantly higher than the 46.00 ± 20.93 years observed for dermatomyositis-related deaths in which neoplasm was not mentioned (p = 0.0000). However, no statistical differences were observed between polymyositis-related deaths with a joint mention of neoplasm and those without in terms of the mean age at death, which was 61.05 ± 15.81 and 53.78 ± 18.00 years, respectively (p = 0.0744). The principal sites of the malignant neoplasms were the digestive tract, breasts, ovaries and lungs, as well as the lymphoid, hematopoietic and related tissues. There was one death in which polymyositis was mentioned jointly with polycythemia vera, a neoplasm classified as being of uncertain or unknown behaviour (Table 4)

**Table 4:** Variables (n (%)) related to neoplams jointly listed with dermatomyositis and polymyositis in death certificates, state of São Paulo, Brazil, 1985 to 2007

Variables	Dermatomyositis	Polymyositis	Total
NEOPLAMS AS AN CAUSE OF DEATH			
Underlying	13 (36.1)	0 (0.0)	13 (23.2)
Associated (Non-underlying)	23 (63.9)	20 (100.0)	43 (76.8)
Total	36 (100.0)	20 (100.0)	56 (100.0)
STANDARDIZED MORTALITY RATES (PER 1 000 000 POPULATION)			
Value	0.0446	0.0249	0.0695
Confidence interval ± 95%	0.0300-0.0591	0.0140-0.0359	0.0512-0.0877
GENDER			
Male	8 (22.2)	8 (40.0)	16 (28.6)
Female	28 (77.8)	12 (60.0)	40 (71.4)
Total	36 (100.0)	20 (100.0)	56 (100.0)
Ratio F:M	3.5:1	1.5:1	2.5:1
AGE AT DEATH (YEARS)			
Mean (Standard deviation)	61.50 (± 13.66)	61.05 (± 15.82)	61.34 (± 14.38)
Median	64.50	64.50	64.50
Mode	50.50	54.50	50.50
SITES (CID-9) (CID-10)			
Digestive organs (150-159) (C15-C26)	9 (25.0)	3 (15.0)	12 (21.4)
*Stomach (151.9) (C16.9)*	*2 (5.6)*	*0 (0.0)*	*2 (3.6)*
*Duodenum (152.0)*	*1 (2.8)*	*0 (0.0)*	*1 (1.8)*
*Colon (C18.9)*	*1 (2.8)*	*1 (5.0)*	*2 (3.6)*
*Rectum (154.1)*	*1 (2.8)*	*0 (0.0)*	*1 (1.8)*
*Intestine (159.0)*	*1 (2.8)*	*0 (0.0)*	*1 (1.8)*
*Unspecified digestivo organ (159.9)*	*0 (0.0)*	*1 (5.0)*	*1 (1.8)*
*Billiary tract (C24.9)*	*1 (2.8)*	*0 (0.0)*	*1 (1.8)*
*Spleen (C26.1)*	*1 (2.8)*	*0 (0.0)*	*1 (1.8)*
Breast (174) (C50)	8 (22.2)	4 (20.0)	12 (21.4)
Female genital organs (179-189) (C51-C58)	7 (19.4)	1 (5.0)	8 (14.3)
*Cervix uteri (180.9) (C53.9)*	*2 (5.6)*	*1 (5.0)*	*3 (5.4)*
*Ovary (183.0) (C56)*	*5 (13.9)*	*0 (0.0)*	*5 (8.9)*
Lung (162.9) (C34)	4 (11.1)	2 (10.0)	6 (10.7)
Lymphoid, haematopoetic and related tissues (200-208) (C81-C96)	3 (8.3)	3 (15.0)	6 (10.7)
*Hodgkin's disease (C81.9)*	*0 (0.0)*	*1 (5.0)*	*1 (1.8)*
*Non-Hodgkin's lymphoma (202.8) (C85.9)*	*3 (8.3)*	*0 (0.0)*	*3 (5.4)*
*Myeloid leukaemia (C92.1)*	*0 (0.0)*	*1 (5.0)*	*1 (1.8)*
*Polycythaemia vera (D45)*	*0 (0.0)*	*1 (5.0)*	*1 (1.8)*
Urinary tract (188-189) (C64-C68)	2 (5.6)	2 (10.0)	4 (7.1)
*Kidney (189.0)*	*0 (0.0)*	*1 (5.0)*	*1 (1.8)*
*Bladder (C67.9)*	*2 (5.6)*	*1 (5.0)*	*3 (5.4)*
Other	3 (8.3)	5 (25.0)	8 (14.3)
*Base of tongue (C01)*	*1 (2.8)*	*0 (0.0)*	*1 (1.8)*
*Prostate (185.9)*	*0 (0.0)*	*1 (5.0)*	*1 (1.8)*
*Kaposi's sarcoma (C46.9)*	*0 (0.0)*	*1 (5.0)*	*1 (1.8)*
*Abdomen (195.2)*	*0 (0.0)*	*1 (5.0)*	*1 (1.8)*
*Malignant neoplasm without specification of site (199) (C80)*	*2 (5.6)*	*2 (10.0)*	*4 (7.1)*
Total	36 (100.0)	20 (100.0)	56 (100.0)

Source: Fundação Sistema Estadual de Análise de Dados (SEADE)

## Discussion

The present study revisits the issue of mortality related to dermatomyositis and polymyositis 25 years after the publication of the only analogous study on the topic [39] and 37 years after mortality related to dermatomyositis was first studied in the United States [7]. The use of multiple cause of death analysis is one of the principal characteristics and advantages of the present study. The multiple cause of death analysis method was employed in order to identify the greatest possible number of deaths related autoimmune diseases, which are frequently mentioned as associated causes of death [16]. This occurred in the present study, in which dermatomyositis or polymyositis was classified as an associated cause in 44.8% of the deaths. The underestimation of chronic rheumatic diseases in mortality statistics has been attributed to the identification of one of their complications, such as an acute process, inflammation, infection or cardiovascular diseases, that frequently becomes the underlying cause of death [5,6]. Specifically, the underestimation of dermatomyositis and polymyositis, understood as associated causes of death, has been attributed to their association with malignant neoplasms [39].

The example of the underestimation of rheumatic diseases in underlying-cause mortality statistics has been employed as an argument for the broad use of multiple cause of death analysis. The tabulation of half of the deaths in the United States in 1955 revealed that approximately 90% of the causes of death included in the categories of arthritis and other rheumatic diseases were not considered in the primary statistics, in which rheumatoid arthritis was underestimated by approximately 85% and other types of arthritis were underestimated by more than 90%, representing the extreme of underestimation, in contrast with neoplasm, which was underestimated by only 10% [7,40,41]. A study conducted in Oxford, England evaluated the influence that mortality coding rules and the introduction of automatic data processing have had on mortality statistics [42]. The authors found that rheumatoid arthritis and other inflammatory polyarthropathies were underestimated by 75.5%, 53.7% and 73.3% in the 1978-1983, 1984-1992 and 1993-1998 periods, respectively. In France, the number of deaths related to rheumatoid arthritis in the 1970-2002 period nearly tripled when all death-certificate mentions of the disease were considered [17].

Over the 23-year period evaluated in the present study, the dermatomyositis-related mortality rate did not vary significantly, whereas the mortality rates for polymyositis as the underlying cause and in terms of total mentions decreased, this decrease being attributed in part to a drop in the mortality rates for total mentions of polymyositis in men. There are currently no parameters for the evaluation of the magnitude and behavior of dermatomyositis- and polymyositis-related mortality. Therefore, we will draw comparisons with the studies conducted in the United States, notwithstanding the limitations regarding mortality statistics, the structure of the respective populations and the time difference between the compared periods. For dermatomyositis as the underlying cause of death, the mortality rates for men and women in the United States were, respectively, 0.3 and 0.6 per 1,000,000 population in the 1959-1961 period [7], higher than the 0.10 and 0.35 per 1,000,000 population we determined for the state of São Paulo in the 1985-2007 period. However, the study of the mortality rates for dermatomyositis and polymyositis as the underlying cause of death in the United States between 1968 and 1978 showed only gender- and race-specific mortality rates [39]. Therefore, the mortality rate for the general population in that period was recalculated, resulting in a crude mortality rate of 1.55/1,000,000 population, also higher than the 0.44/1,000,000 population we observed for the state of São Paulo in the study period.

In the present study, the standardized dermatomyositis- or polymyositis-related mortality rates were consistently higher for women than for men, a finding that is consistent with the greater proportion of women reported in previous studies on mortality [4,39], as well as in studies involving patients with dermatomyositis or polymyositis [1,3,8,37,38,43-47]. The higher dermatomyositis- and polymyositis-related mortality among women is related to higher prevalence of the diseases in the female gender.

Our observation that the highest specific mortality rates were among older individuals is consistent with the results of the studies conducted in the United States and in France [39,48]. In dermatomyositis and polymyositis, age is considered one of the factors that are prognostic of lower survival and a greater risk of death. The finding that the age at death was lower among dermatomyositis-related deaths than among polymyositis-related deaths might be due to the fact that dermatomyositis has an earlier onset [37,47]. However, the temporal increase in the mean age at death among women who died from polymyositis is primarily due to the increase in life expectancy for women in the state of São Paulo, which rose from 70.02 to 77.98 years between 1980 and 2007.

Mortality statistics, like the used in this study, suffer from two types of limitations: quantitative and qualitative. In the State of São Paulo, the quantitative limitations relating to the coverage of the number of deaths that occur are unimportant. The relationship between the deaths informed to SEADE Foundation and those estimated by means of demographic projections available for recent years shows figures even above 100%, which suggests that the mortality data coverage is very good [49]. With regard to the qualitative limitations in this State, ill-defined causes were considered to be the underlying cause of death in only 6.11% and 6.41%, in relation to the total number of deaths for the 1985-1995 and 1996-2007 periods, respectively--values that are lower by half than those reported for the country as a whole [50-52]. Furthermore, no difficulties were presented in transforming the causes of death into ICD codes, among the death certificates mentioning dermatomyositis and polymyositis. It has been seen that the coding work was accurate and did not constitute a source of error that might have compromised the quality of the mortality statistics [18]. The processing of the causes of death at the SEADE Foundation was performed in accordance with the recommendations of the WHO for the identification of the underlying cause of death, and there was rigorous quality control of the automatic data processing. An important limitation refers to the fact of dermatomyositis and polymyositis not being recorded anywhere by physicians that complete death certificates of deceased patients. To our knowledge this problem has not been studied to date for these causes of death. Notwithstanding, the lack of attention to mortality outcomes has been justified by the argument that death certificates of patients with chronic rheumatic diseases do not mention rheumatic diseases at all [6].

One of the principal resources derived from the use of multiple cause of death analysis is the recovery of data related to the associated causes of death listed in the medical certification section of the death certificate. Greater understanding of the natural history of myopathies translates to increased patient survival [44]. Knowledge of the associated causes, resulting from concomitant diseases [38], opens new perspectives for the prevention of death primarily attributed to dermatomyositis or polymyositis. Pneumonia, which is one of the principal associated causes, is particularly important due to the fact that its severity increases when it occurs jointly with septicemia, as shown in the present study. The factors predisposing to infectious complications include esophageal, muscular and pulmonary involvement, as well as the use of immunosuppressants [53]. In France, infectious complications were found to be principal immediate cause of death in patients with dermatomyositis or polymyositis, occurring in approximately 45.8% [54]. However, despite the fact that, on death certificates in Brazil, respiratory failure is frequently listed as an immediate cause of death [52], we found that it was often mentioned jointly with pneumonia. Interstitial lung disease and related conditions occurred with greater frequency in dermatomyositis than in polymyositis. Together, pneumonia, respiratory failure and interstitial lung disease/related conditions represent the greatest risk of death in patients with septicemia, and respiratory impairment has been reported to occur in up to 70% of such patients [4,55], the lungs being the organ system most often affected by diseases accompanying myopathies [38].

We also found that circulatory diseases were common associated causes of death--heart failure, arrhythmias, arterial hypertension, coronary ischemia and cardiorespiratory arrest prevailing as the most common immediate causes of death. These findings are consistent with reports that up to 75% of patients with myositis present cardiovascular involvement [56,57].

Table 4 presents the underlying causes of death for the deaths in which dermatomyositis and polymyositis were identified as associated causes. Considering the chapters of the ICD, neoplasm, diseases of the respiratory system and diseases of the circulatory system, together with certain infectious diseases and parasitic diseases, predominate. Many of the underlying causes of death included in Table 4 were also listed as associated causes, an eventuality that depends greatly on the weight that the physician gives to a particular condition, on the way in which the medical certification section of the death certificate is filled out and on the WHO guidelines and mortality standards regarding the condition. These considerations primarily apply to conditions inherent to the natural history of dermatomyositis and polymyositis. Chief among the deaths from infectious diseases were those attributed to tuberculosis and those attributed to AIDS. Characteristic of the overlap syndrome, there were deaths in which the death certificate listed systemic lupus erythematosus or systemic sclerosis as the underlying cause of dermatomyositis or polymyositis.

The mean number of causes of death listed per death certificate constitutes one of the principal indicators regarding multiple cause of death analysis [14,15,18]. In the present study, the consideration of only the underlying cause of death would have resulted in the loss of approximately two causes of death per death certificate. The lower mean number of causes on the death certificates on which dermatomyositis or polymyositis was listed as the underlying cause is attributable to the greater proportion of death certificates listing only one or two causes, unlike the death certificates listing dermatomyositis or polymyositis as associated causes, on which three or four causes were needed in order to describe the underlying cause. However, the greater mean number of causes in the 1996-2007 period is primarily due to the new line (d) introduced into the medical certification section of the death certificate when the ICD-10 entered into effect, as well as to better completion of the death certificates by physicians [13,18]. Nevertheless, the mean number of causes listed on the death certificates related to deaths from dermatomyositis or polymyositis, except for those on which dermatomyositis was listed as the underlying cause, is higher than that found for the state of São Paulo or for Brazil as a whole, which was 3.15 ± 1.37 and 2.81 ± 1.38, respectively, in 2003 [18].

The greater risk of death linked to dermatomyositis accompanied by neoplasm was consistent with the results of an earlier study [58], in which the mortality data were decisive in elucidating the risk of neoplasm in patients with dermatomyositis [34,58,59]. In addition, we found that, among the deaths related to dermatomyositis, the mean age at death was higher in those with a joint mention of neoplasm that in those in which neoplasm was not mentioned. This was also observed in patients in Spain, Hungary and France, where the mean and median ages, as well as the sites of the neoplasms, were quite similar to those found in the present study [47,48,60-62].

## Conclusions

In the present study, we have used multiple cause of death analysis to describe the characteristics of dermatomyositis- and polymyositis-related mortality over the course of the 23-year study period (1985-2007). We find it worrisome that dermatomyositis or polymyositis was identified as the underlying cause of death in only 55.2% of the deaths studied. This indicates that both diseases were underestimated in the primary mortality statistics. We also observed a predominance of deaths in women and in older individuals, as well as a trend toward stability in the mortality rates, except for a drop observed in the mortality rates for polymyositis in men, as the underlying cause and in terms of the total number of mentions. The investigation of the underlying and associated causes of death related to dermatomyositis or polymyositis broaden the knowledge of the natural history of both diseases. We have confirmed that the risk of death is greater when either disease is accompanied by neoplasm, albeit to lesser degree in individuals with polymyositis. The results of the present study could help integrate mortality data for use in the evaluation of control measures for dermatomyositis and polymyositis.

## Competing interests

The authors declare that they have no competing interests.

## Authors' contributions

AHS participated in the design of the study, processing, analysis and interpretation of data and drafted the manuscript, JMPS performed the regression analysis, CEP developed all software systems to process multiple cause-of-death, DCCS and EIS revised the manuscript. All authors read and approved the final manuscript.

## Pre-publication history

The pre-publication history for this paper can be accessed here:

http://www.biomedcentral.com/1471-2458/10/597/prepub
